# Granular cell tumor mimicking breast carcinoma in a 49-year-old female: a case report

**DOI:** 10.3389/fmed.2026.1810784

**Published:** 2026-07-15

**Authors:** Fadoua El Mansouri, Khadija Bichri, Zakaria Hafiani, Mohammed Zarqaoui, Bouchra Ghazi

**Affiliations:** 1 Immunopathology-Immunotherapy-Immunomonitoring Laboratory, Faculty of Medicine, Mohammed VI University of Sciences and Health (UM6SS), Casablanca, Morocco; 2 Moulay Youssef Breast Center, Casablanca, Morocco; 3 Department of Obstetrics and Gynecology, Les Iris Clinic, Casablanca, Morocco; 4 Reproductive Health Physiopathology Laboratory, Mohammed IV Center for Research and Innovation (CM6RI), Rabat, Morocco; 5 Department of Obstetrics and Gynecology, Mohammed VI International University Hospital, Bouskoura, Morocco

**Keywords:** Abrikossoff tumor, breast cancer, carcinoma mimic, granular cell tumor, immunohistochemistry

## Abstract

Abrikossoff entities, also known as granular cell tumors (GCTs), are categorized as rare benign soft-tissue tumors of Schwann cell origin. The most common site of predilection is the oral cavity, followed by the skin, breast, digestive tract, and tracheobronchial tree; the least common location is the central nervous system. They are benign growths associated with a good prognosis, with surgical resection being their standard treatment. However, sometimes, they become malignant. They typically manifest as solitary tumors; hence, multiple lesions are rare. Here, we report a 49-year-old woman with a palpable and painful mass in her right breast. While the clinical and radiological features favored a diagnosis of epidermoid cyst, immunohistochemistry confirmed an Abrikossoff tumor. No recurrence of the GCT was assessed during the control.

## 1 Introduction

Abrikossoff's tumor, also known as a granular cell tumor (GCT), is an infrequent condition that occurs in soft tissues (1). Rarely malignant, this mass is most commonly seen in the oral cavity and was first described in the tongue in 1926 (2). However, this benign tumor can also occur in other locations, including the skin, breast, salivary glands, vocal cords, larynx, gastrointestinal tract, genital tract, and respiratory tract (3). GCTs happen to occur in the breast with a rate of 1 per 1,000 breast tumors, and account for 5 to 15% of all the registered cases of GCTs (4, 5). Breast cancer is the most diagnosed malignancy, and one of the leading causes of mortality in females (6). According to the global cancer statistics of 2022, the incidence rate of this disease was of 11.6% in the world, and its death rate was of 6.9% (7). The apparition of a lump, abnormality of the nipple, and manifestation of pain are alarming symptoms that raise the suspicion of the occurrence of a breast tumor (8). Even if advancements in early detection and therapeutic strategies have improved outcomes, breast cancers still exhibit a complex pathophysiology requiring a comprehensive understanding of their molecular mechanisms (9).

The immunohistochemical study led by Stewart revealed that Schwann cells are the origin of GCT, with tissues showing strong expression of the neurogenic marker S-100, a calcium-binding protein subfamily (10). GCT in the breast is often misdiagnosed as breast cancer, with definitive diagnosis based on immunohistochemical examination. The only treatment for Abrikossoff tumor is surgical excision (11).

In this case report, we present a 49-year-old female with a GCT located in the right breast, which was confused with an epidermoid cyst during initial clinical examination. Abrikossoff's tumor was confirmed by histopathological and immunohistochemical analysis.

## 2 Case description

In 2020, a 49-year-old Moroccan female, having undergone a thyroidectomy and taking birth control since the age of 16, assessed a painful mass on her right breast. The appearance of the entity suggested that the lesion was benign, which guided our clinical decision-making. The absence of breast magnetic resonance imaging was due to the limited resources in the treating institution.

At the first consultation, the patient exhibited a breast abscess of 15 mm in diameter, which led us to think of a bacterial infection. As no microbiological documentation was provided, empirical antibiotic therapy started with pyostacine, combined with local antisepsis using hexomidine, to guarantee aseptic conditions before surgical intervention. No improvement was noted. On September 3^rd^, 2020, the preoperative assessment included complete blood count and blood typing. On September 22^nd^, 2020, a tumorectomy was performed under local anesthesia with sedation, in the right breast. The surgery consisted of an elliptical incision with a skin collar, complete tumor removal, hemostasis, and layered closure. Given the suspicion of a benign epidermal cyst, only the excisional surgery was accomplished, without prior percutaneous biopsy. Furthermore, the absence of core-needle biopsy equipment at the institution excluded this option.

**Figure 1:**
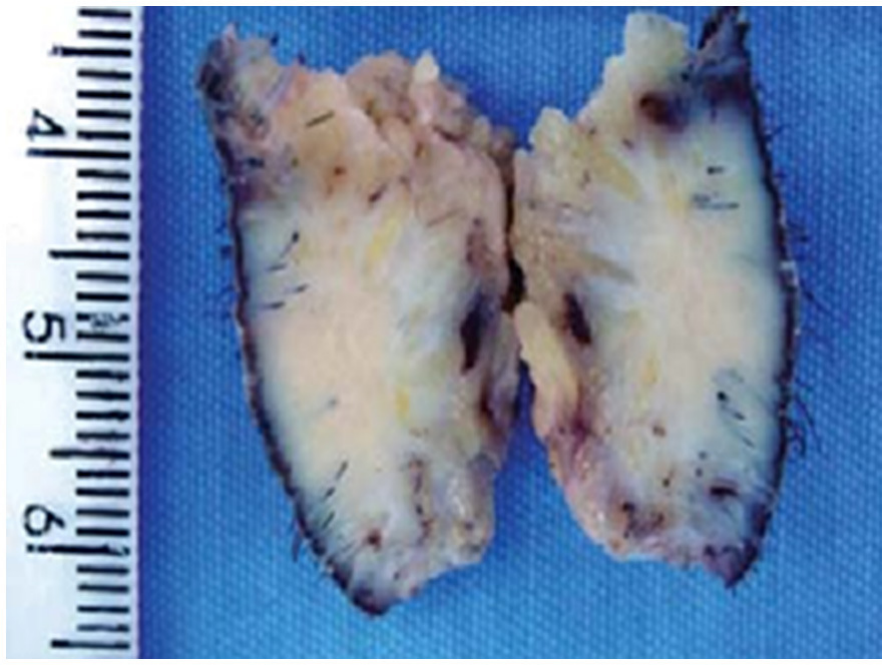
Macroscopic aspect of a poorly defined, stellate, whitish tumor with a firm to hard consistency (anchored borders).

**Figure 2:**
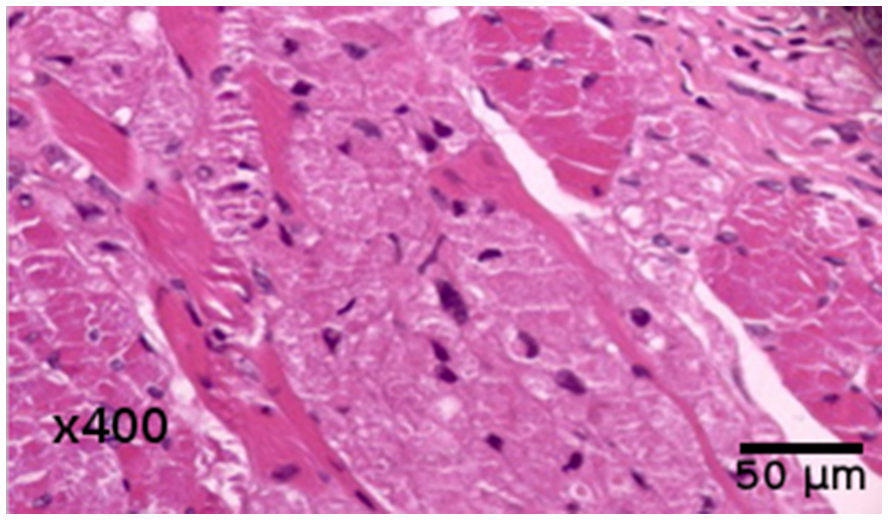
Breast proliferation of cells, with cords of granular cells with mild nuclear (hematoxylin, x400).

After examination of the excision specimen, negative surgical margins measured approximately 2–3 mm. The resected specimen weighed 10g and measured 3 x 1.5 cm. Its macroscopic examination exhibited a friable, beige-colored process. The examination revealed a benign Abrikossoff's tumor, with a height of 32 mm, bordered by margins of 0.5 cm and 0.3 cm of mammary parenchyma with adipose regression (Figure 1). The performance of total inclusion allowed the histological analysis of the totality of the specimen. The microscopical exam revealed a mix of fibroadipose tissue punctuated with lymphocytes and a proliferation of irregular cords of cells. The cells were round or polyhedric with abundant granular eosinophilic cytoplasm. The nuclei were ovoid and devoid of abnormal mitoses, with a low mitosis index and no necrosis (Figure 2). Furthermore, cell-lobulating collagen trabeculae were assessed, with no surface epithelium.

In immunohistochemistry, S-100 was strongly expressed in the tumor tissue (Figure 3A). Furthermore, CD68 was strongly positive in tumor cells, with levels decreasing at the periphery of the resection site, indicating tumor mass removal (Figure 3B). Staining with the pan-cytokeratin antibody (AE1/AE3) was negative, and Ki67 was expressed in less than 1% of tumor cells. The diagnosis relied heavily on the Fanburg-Smith classification. Evaluation of the 6 criteria associated with malignancy, namely necrosis, spindling of tumor cells, vesicular nuclei with large nucleoli, increased mitotic activity, high nuclear-to-cytoplasmic ratio, and nuclear polymorphism, yielded a score of 0 out of 6. These results further confirmed the presence of a benign GCT.

**Figure 3A:**
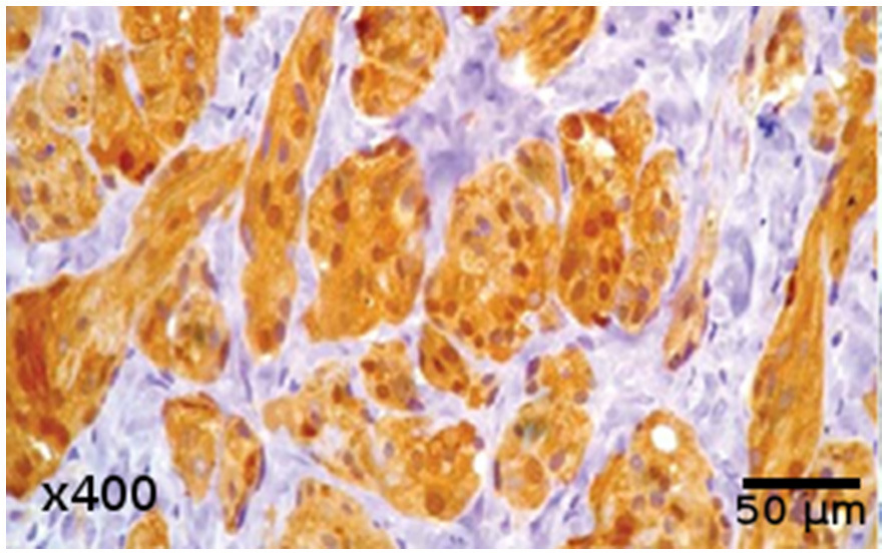
Expression of S-100 in granular cells (Thermo-Fisher cloning solution, hematoxylin, x400).

**Figure 3B:**
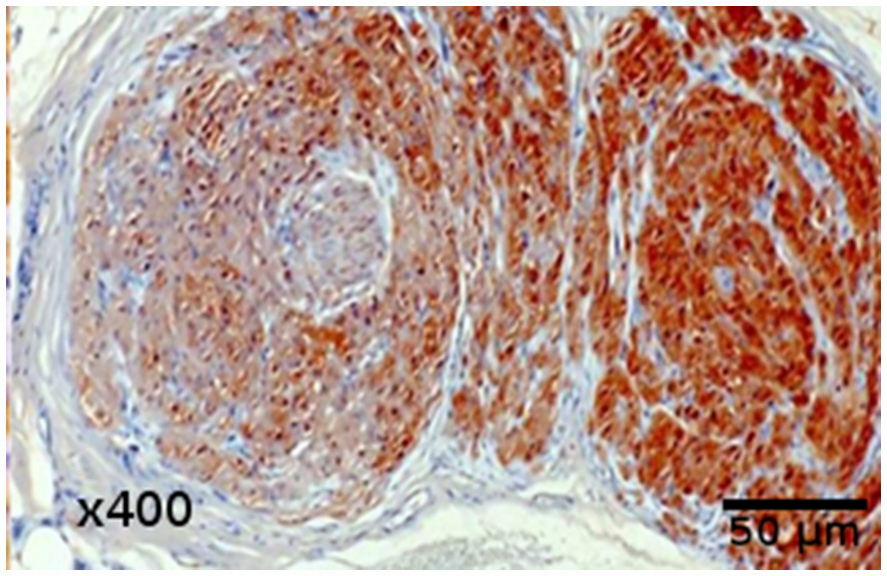
Expression of CD68 in granular cells (hematoxylin, x400).

As Abrikossoff's tumor is benign, only surgical resection was completed. No treatment was adopted, apart from drugs for better wound healing. A silicone scar tape CEREDERM 5x8 cm was proposed for the scar, along with the application of BETNEVAL cream (two local applications per day).

A follow-up mammogram in 3 years showed two simple bilateral cysts at the junction of the superior quadrants of the right breast, contiguous, measuring 3.5 mm and 4 mm in diameter. On the other hand, the left breast upper outer quadrant showed a simple cyst of 10.5 mm, associated with a microcyst of 2.5 mm. No tissular glandular lesion or architectural disorganization was assessed. Both cysts were categorized as BIRADS2. No diagnostic challenges were identified. The following table retells the historical and clinical timeline of the patient (Table 1).

**Table 1:** Timeline of historical and current information about the patient.

**August 2020**	Intake of pyostacine and hexomidine after assessment of the painful mass in the right breast, no amelioration was assessed.
**September 2020**	Surgical resection and close follow-up of the patient's condition.
**September 2020**	Histological examination revealed a benign tumor, namely an Abrikossoff tumor, with a height of 32 mm.
**January 2022**	Mammography revealed a simple cyst in the superior external quadrant of the left breast (26 mm x 10 mm). Absence of benign abnormalities.
**September 2022**	Echography revealed a simple cyst in the superior external quadrant of the left breast (28 mm x 11 mm). Absence of benign abnormalities.
**May 2023**	Mammography revealed the disappearance of the cyst in the superior external quadrant of the right breast and the presence of small, simple bilateral cysts, with no abnormalities. Both cysts were categorized as BIRADS2. No abnormality in the right breast either.

As the authors were not involved in the patient's clinical management, the reconstruction of the diagnosis and the therapy was based on available medical records. Therefore, documentation regarding the initial diagnosis and surgical decision was not available in the source data.

## 3 Discussion

The first case of GCT was confused with myoblastic myoma (2). However, the detection of S-100 protein in tumor cells suggested a neurogenic origin of the disease. Indeed, histochemical examination and electron microscopy showed that Schwann cells were at the origin of GCT (12). These tumors can generate in soft tissues, mucosal sites, and skeletal muscle (13–16). GCT has a higher incidence rate in the mature population, even though the young population is also at risk (4, 15, 17, 18). Often described as solitary neoplasms, but possibly also being multiple, GCTs have been associated with neurofibromatosis, Noonan syndrome, and LEOPARD syndrome (3, 19, 20). Our case study didn't present any of these syndromes and presented a solitary neoplasm.

GCT in the breast can arise in the breast parenchyma, but the most common location is the upper inner quadrant, along the supraclavicular nerve (5, 21). Over time, other locations were found to hold GCT, including the upper outer quadrant, axillary region, and nipple, and could mimic breast carcinoma (Table 2) (22–24). Our patient has a GCT in the upper inner quadrant of the right breast. A small number of patients are asymptomatic. However, symptomatic patients may present with discomfort, pruritus, skin retraction, skin thickening, dimpling, and reactive lymphadenopathy (25). Our case presented pain in the tumor. Several factors led to the misdiagnosis of this GCT. First, the suspicion of a benign epidermoid cyst; second, no further investigation was carried out as part of the workup. On reflection, imaging modalities such as ultrasound or MRI would have been beneficial, providing a more thorough characterization of the mass before surgical intervention. Thus, it would have revealed potential discrepancies with the epidermoid cyst.

**Table 2:** Criteria differentiating between GCT and breast cancer.

Criteria	Granular cell tumor	Breast cancer
Imaging	Spiculated margins, with well to ill-defined margins, significant depth, and posterior acoustic shadowing (3).	Characterized by irregular shape and ill-defined borders, microcalcifications, spiculated margins, or distorted masses (4, 5).
Histopathology	Neoplasms originating from Schwann cells. Tumor cells are round or polygonal with profuse granular eosinophilic cytoplasm, organized in nests and sheets in collagenous stroma. Nuclei are central and can either be small and dark or large with vesicular chromatin (6).	Proliferation of epithelial cells within the breast milk ducts, restricted by the basement membrane without invading adjacent tissues (7, 8).
Immunochemistry	Positive staining for S100, CD68, neuron-specific enolase (NSE), CD57, CD68, vimentin, calretinin, PGP9.5, SOX10, CD56, and inhibin (9). Negative staining for progesterone and estrogen receptors, actin -α smooth muscle, CD31, CD34, CD105, and desmin (3).	Staining for estrogen receptor, progesterone receptor, human epidermal growth factor receptor 2, and Ki-67 (10).

To evaluate a suspicious solid breast mass, the standard procedure typically indicates a preoperative tissue diagnosis (by using a core-needle biopsy) along with the use of appropriate imaging. In this regard, breast magnetic resonance imaging is a much-appreciated tool, but its absence is a significant limitation. This procedure greatly helps guide the surgery and avoid any unnecessary aggressive strategy. In our case, this deficit in imaging materials highlights the importance of optimizing diagnostic strategies within existing current obstacles. In the case of benign GCT, a simple excision with negative margins is considered sufficient. Literature recommends a safety margin of >10 mm, as complete excision can reach only a rate of 2 to 8% risk of recurrence, while incomplete excision can reach 21 to 50 % of recurrence rate (26, 27). Moreover, studies have shown that GCT poses little long-term risk of recurrence, despite excision with positive margins (28). With clear surgical margins of 2–3 mm and a low risk of recurrence related to GCTs, we were further encouraged to adopt postoperative follow-up rather than re-excision.

In the current study, the diagnostic and therapeutic pathways were significantly affected by limited resources. The surgical excision of a presumed epidermoid cyst resulted from the lack of core needle biopsy equipment. In the same way, the unavailability of advanced imaging modalities, for instance, MRI, illustrates the diagnostic obstacles encountered in limited-resource settings. The restrictions resulted in surgical margins of 2 and 3 mm, which fell below the recommended threshold of >10 mm for GCT (27). Regardless, no further surgery was planned, and the patient was followed clinically in accordance with the literature recommendation of close follow-up rather than re-intervention for benign tumors with clear but small margins (29). Therefore, this case exemplifies how resource limitations can directly impact surgical planning and recovery management, and exacerbates the need for tailored clinical protocols in situations where standard diagnostic tools are lacking.

At the macroscopic level, GCTs are characterized by a pale creamish to grayish-brownish color, with well to ill-defined limits (30). They can also be covered by a gray-white mucosa and appear as solid tumors with a faded pink color and a granular appearance (31, 32). In our case, the solid tumor appeared beige, with a dark mucosa and a granular appearance (Figure 3).

Molecular profiling of GCT revealed the importance of ATP6A1 and ATP6A2 in Schwann and epithelial cells, as their *in vitro* silencing led to the apparition of an eosinophilic granular cytoplasm, along with the acquisition of oncogenic criteria via PDGFR-β, Src-family kinases, and STAT5 signaling (33). Additionally, GCT is positive for S100 protein, CD68, vimentin, calretinin, PGP9.5, neuron-specific enolase, CD57, SOX10, CD56, and inhibin, whereas it is negative for progesterone and estrogen receptors (34–40). The patient studied here showed positive expression for S100 and CD68 (Figures 3A extbfand 3B). Additionally, the tumor was negative for the pan-Cytokeratin (AE1/AE3) antibody, and less than 1% of tumor cells expressed Ki-67.

The cytological study of GCT revealed the round shape and small size of the nuclei, their central position, and dense chromatin (41, 42). Sometimes, the cells can be binucleated and found in a deeply proteinaceous material (43). Moreover, cells are polygonal and contain eosinophilic lysosomes, which are responsible for the granular appearance of the tumor. Although rarely round, the cells can adopt a spindle-like shape, with accumulation of the same type of cytoplasm (44). To distinguish benign from malignant GCT, Fanburg-Smith et al. proposed a classification based on necrosis, spindling, vesicular nuclei, large nucleoli, pleomorphism, high nuclear-to-cytoplasmic ratio, and a mitotic coefficient greater than 2 per 10 fields. Indeed, finding three or more of these characteristics in a lesion associates it with malignant neoplasm (45). They are also represented as a nonencapsulated accumulation of elongated eosinophilic cells, with granular content (46). Our case showed polygonal cells with granular cytoplasm, with no mitosis, necrosis, or atypia (Figure 2). The benign character of this lesion was evaluated based on the Fanburg-Smith classification, as no typical feature of malignancy was observed.

Histologically, GTCs are well-delimited, with a nest-like and sheet-like appearance of polygonal cells with clear borders and a profuse granular eosinophilic cytoplasm, and are interconnected by bands of connective tissue (5). Occasionally, Pustulo-ovoid bodies of Milian are assessed. These bodies are large granules, surrounded by a clear halo (47). In our study, we noted polygonal cells with eccentric hyperchromatic nuclei. Some Pustulo-ovoid bodies of Milian are also noticed (Figure 2).

The importance of differentiating between breast carcinoma and GCT of the breast resides in the different treatments that each case needs. Indeed, to avoid extensive resection of carcinoma, it is primordial to include GCT in the diagnosis for breast lesions. Our case presented a benign tumor that was completely removed. In this patient's follow-up, no recurrence has been observed after 3 years.

Several limitations were encountered in this case report, including the lack of additional clinical cases and the authors' absence from the patient's clinical management. Moreover, the analysis was based on retrospective medical records, and the documentation lacked an imaging strategy due to limited imaging resources at the treating institution. The molecular profiling was not available, and no breast magnetic resonance imaging was performed. These limitations reveal real-world hindrances of retrospective case reporting and are acknowledged transparently.

Given the low incidence of GCT and its limited characterization, this case enriches the limited body of literature on this tumor. Moreover, GCTs are usually described as benign tumors able to mimic carcinoma. However, in the current study, this tumor mimics a benign epidermoid cyst, which is rarely reported in the literature (26). Therefore, this case is a valuable addition to existing literature. As such, the incidental finding of this tumor after the excision of a mass clinically and radiologically related to an epidermoid cyst shows a diagnosis not previously well documented. The histopathological results, which included a friable, beige-colored process measuring 3.2 cm with hemorrhagic changes, and the need for complementary immunohistochemical analysis to clarify the final diagnosis, further confirm the existing pathological description of this entity. This case demonstrates GCT's ability to mimic a clinically common benign lesion, such as an epidermoid cyst, thereby increasing diagnostic complexity.

This case presents interesting lessons for clinicians performing in resource-limited or well-equipped settings. First, GCTs are commonly reported in the literature to mimic carcinomas. However, our case showed an atypical resemblance to a benign epidermoid cyst. This highlights the necessity of clinicians to remain careful, even when clinical and radiological features point toward benignity, especially when there are concurrent infectious signs such as a breast abscess. Second, a core needle biopsy should be performed, if possible, for preoperative histological confirmation. This procedure allows adaptation of the surgical plan and reduces the risk of unplanned excision with insufficient margins. Third, the unavailability of standard diagnosis tools should be documented and explicitly mentioned in the recovery management strategy, involving clinical follow-up. Lastly, histological examination, with total inclusion of the surgical specimen, is necessary to shun misdiagnosis of rare benign entities mimicking benign conditions. In the current case, the complete inclusion of GCT proved decisive in establishing the final diagnosis.

## 4 Conclusion

The rate of occurrence of Abrikossoff's tumor is low; it is important to detect it properly to avoid confounding it with breast carcinoma. Imaging techniques are insufficient to detect it, whereas core needle biopsy and immunochemistry are crucial for a bona fide diagnosis. Clinicians need to integrate the assessment of both malignant and benign neoplasms to avoid misdiagnosis and unnecessary surgical resection. Therefore, this case report highlights the need for appropriate imaging and tissue diagnosis, as well as the potential for overtreatment when standard diagnostic pathways are deviated from.

## Data Availability

The original contributions presented in the study are included in the article/supplementary material, further inquiries can be directed to the corresponding author.

## References

[1] Ardeleanu V Jecan RC Moroianu M Teodoreanu RN Tebeica T Moroianu LA . Case report: Abrikossoff's tumor of the facial skin. Front Med. (2023) 10:1149735. doi: 10.3389/fmed.2023.1149735 37324160 PMC10264634

[2] Abrikossoff A Über Myome. Virchows Arch path Anat. (1926) 260:215–33. doi: 10.1007/BF02078314

[3] Neelon D Lannan F Childs J. Granular Cell Tumor. In: StatPearls. Treasure Island (FL): StatPearls Publishing (2025). Available online at: http://www.ncbi.nlm.nih.gov/books/NBK563150/ [Accessed 2025 May 14].

[4] Lack EE Worsham GF Callihan MD Crawford BE Klappenbach S Rowden G . Granular cell tumor: a clinicopathologic study of 110 patients. J Surg Oncol. (1980) 13:301–16. doi: 10.1002/jso.2930130405 6246310

[5] Adeniran A Al-Ahmadie H Mahoney MC Robinson-Smith TM. Granular cell tumor of the breast: a series of 17 cases and review of the literature. Breast J. (2004) 10:528–31. doi: 10.1111/j.1075-122X.2004.21525.x 15569210

[6] Contiero P Boffi R Borgini A Fabiano S Tittarelli A Mian M . Causes of death in women with breast cancer: a risks and rates study on a population-based cohort. Front Oncol. (2023) 13:1270877. doi: 10.3389/fonc.2023.1270877 38023134 PMC10646497

[7] Bray F Laversanne M Sung H Ferlay J Siegel RL Soerjomataram I . Global cancer statistics 2022: GLOBOCAN estimates of incidence and mortality worldwide for 36 cancers in 185 countries. CA Cancer J Clin. (2024) 74:229–63. doi: 10.3322/caac.21834 38572751

[8] Koo MM von Wagner C Abel GA McPhail S Rubin GP Lyratzopoulos G. Typical and atypical presenting symptoms of breast cancer and their associations with diagnostic intervals: evidence from a national audit of cancer diagnosis. Cancer Epidemiol. (2017) 48:140–6. doi: 10.1016/j.canep.2017.04.010 28549339 PMC5482318

[9] Xiong X Zheng LW Ding Y Chen YF Cai YW Wang LP . Breast cancer: pathogenesis and treatments. Sig Transduct Target Ther. (2025) 10:49. doi: 10.1038/s41392-024-02108-4 39966355 PMC11836418

[10] Stewart CM Watson RE Eversole LR Fischlschweiger W Leider AS. Oral granular cell tumors: a clinicopathologic and immunocytochemical study. Oral Surgery, Oral Medicine, Oral Pathology. (1988) 65:427–35. doi: 10.1016/0030-4220(88)90357-X 2834681

[11] Musha A Ogawa M Yokoo S. Granular cell tumors of the tongue: fibroma or schwannoma. Head Face Med. (2018) 14:1. doi: 10.1186/s13005-017-0158-9 29329562 PMC5795288

[12] Fisher ER Wechsler H. Granular cell myoblastoma—a misnomer. Electron microscopic and histochemical evidence concerning its schwann cell derivation and nature (granular cell schwannoma). Cancer. (1962) 15:936–954 13893237 10.1002/1097-0142(196209/10)15:5<936::aid-cncr2820150509>3.0.co;2-f

[13] Aoyama K Kamio T Hirano A Seshimo A Kameoka S. Granular cell tumors: a report of six cases. World J Surg Oncol. (2012) 10:204. doi: 10.1186/1477-7819-10-204 23021251 PMC3502223

[14] Li H Zhang M Zheng Y Zhang H. Gastric granular cell tumor: a case report and literature review. Oncol Lett. (2024) 28:1–11. doi: 10.3892/ol.2024.14536 38983126 PMC11228929

[15] Rose B Tamvakopoulos GS Yeung E Pollock R Skinner J Briggs T . Granular Cell Tumours: A Rare Entity in the Musculoskeletal System. Sarcoma. (2009) 2009:765927. doi: 10.1155/2009/765927 20169099 PMC2821775

[16] Arai E Nishida Y Tsukushi S Sugiura H Ishiguro N. Intramuscular Granular Cell Tumor in the Lower Extremities. Clin Orthop Relat Res. (2010) 468:1384–9. doi: 10.1007/s11999-009-1085-2 19760336 PMC2853648

[17] Medina-Morell JR Cheverez-Ocasio JI Negron-Gonzalez V Ramos-Rivera G. Granular cell tumor of the breast in a 17-year-old female: a case report. J Pediatr Surg Case Rep. (2024) 111:102913. doi: 10.1016/j.epsc.2024.102913

[18] Henry M Perry A. Multiple cutaneous granular cell tumors: case report of a 19-year-old African American female. J Cutan Med Surg. (2011) 15:344–6. doi: 10.2310/7750.2011.10088 22202510

[19] Ramaswamy PV Storm CA Filiano JJ Dinulos JGH. Multiple Granular Cell Tumors in a Child with Noonan Syndrome. Pediatr Dermatol. (2010) 27:209–11. doi: 10.1111/j.1525-1470.2010.01111.x 20537083

[20] Aragues IH Domínguez MC Blanco VP Zubicaray BE Fernández RS. LEOPARD syndrome and multiple granular cell tumors: an underreported association? Indian J Dermatol Venereol Leprol. (2016) 82:77. doi: 10.4103/0378-6323.171642 26728819

[21] Gomez M Mehta R. 76 Granular cell tumor, a rare breast tumor. Int J Gynecol Cancer. (2020) 30:A42. doi: 10.1136/ijgc-2020-IGCS.72

[22] Patel A Lefemine V Yousuf SM Abou-Samra W. Granular cell tumour of the pectoral muscle mimicking breast cancer. Cases J. (2008) 1:142. doi: 10.1186/1757-1626-1-142 18775077 PMC2538504

[23] Jung YJ Nam KJ Choo KS Lee K. Granular Cell Tumor of the Axillary Accessory Breast: A Case Report. J Korean Soc Radiol. (2023) 84:275–9. doi: 10.3348/jksr.2022.0129 36818704 PMC9935962

[24] Park SC Kwon YB An SY Ma HU Jung SW Na YM . Granular Cell Tumor of the Male Breast With Nipple Retraction and Pectoralis Major Invasion Treated With Mastectomy: A Case Report. J Breast Dis. (2024) 12:19–22. doi: 10.14449/jbd.2024.12.1.19

[25] Yan J. Granular cell tumor of the breast: A case report and review of literature. World J Clin Cases. (2023) 11:8044–9. doi: 10.12998/wjcc.v11.i33.8044 38075570 PMC10698414

[26] Faenza M Antonetti AM Filosa FG Pelella T Vampa F Pagliuca F . A rare case of granular cell tumor in axillary cavity: an unusual occurrence. J Surg Case Rep. (2026) 2026:rjag099. doi: 10.1093/jscr/rjag099 41993121 PMC13082391

[27] Ferraz PD. Granular cell tumor (Abrikossoff's tumor) of the tongue: A case report. J Cancer Biol. (2021) 2:6–9. doi: 10.46439/cancerbiology.2.016

[28] Papalas JA Wylie JD Dash RC. Recurrence Risk and Margin Status in Granular Cell Tumors of the Breast: A Clinicopathologic Study of 13 Patients. Arch Pathol Lab Med. (2011) 135:1005–1010. doi: 10.5858/2010-0430-OAR.1 21732779

[29] Moniruddin ABM Doly HK Jannat S Hasan T Rouf MA. Granular cell tumors. KYAMC J. (2023) 14:96–101. doi: 10.3329/kyamcj.v14i02.68561

[30] Meves V Pohl J. Esophageal Granular Cell (Abrikossow) Tumor: Macroscopic Appearance and Endoscopic Management (Video). Video J Ency GI Endosc. (2014) 2:87–9. doi: 10.1016/j.vjgien.2015.02.001

[31] Hasanov A Musayev J Onal B Rahimov C Farzaliyev I. Gingival granular cell tumor of the newborn: a case report and review of literature. TJPATH. (2011) 27:161. doi: 10.5146/tjpath.2011.01067 21630205

[32] Kiskinov PI Palavurov AM Mollova-Kyosebekirova AY Atliev KT Zanzov EI Anastasova VN. Unique Case of Rare Non-Neural Granular Cell Tumor of the Rectus Abdominis Muscle. Medicina. (2024) 60:4. doi: 10.3390/medicina60040576 38674222 PMC11051885

[33] Pareja F Brandes AH Basili T Selenica P Geyer FC Fan D . Loss-of-function mutations in ATP6AP1 and ATP6AP2 in granular cell tumors. Nat Commun. (2018) 9:3533. doi: 10.1038/s41467-018-05886-y 30166553 PMC6117336

[34] Abreu N Filipe J André S Marques JC. Granular cell tumor of the breast: correlations between imaging and pathology findings. Radiol Bras. (2020) 53:105–11. doi: 10.1590/0100-3984.2019.0056 32336825 PMC7170582

[35] Le BH Boyer PJ Lewis JE Kapadia SB. Granular Cell Tumor: Immunohistochemical Assessment of Inhibin-α, Protein Gene Product 95, S100 Protein, CD68, and Ki-67 Proliferative Index With Clinical Correlation. Arch Pathol Lab Med. (2004) 128:771–5. doi: 10.5858/2004-128-771-GCTIAO 15214825

[36] Nonaka D Chiriboga L Rubin BP. Sox10: A Pan-Schwannian and Melanocytic Marker. Am J Surg Pathol. (2008) 32:1291–8. doi: 10.1097/PAS.0b013e3181658c14 18636017

[37] Gurzu S Ciortea D Tamasi A Golea M Bodi A Sahlean DI . The immunohistochemical profile of granular cell (Abrikossoff) tumor suggests an endomesenchymal origin. Arch Dermatol Res. (2015) 307:151–7. doi: 10.1007/s00403-014-1505-3 25262119

[38] Fine SW Li M. Expression of Calretinin and the alpha-Subunit of Inhibin in Granular Cell Tumors. Am J Clin Pathol. (2003) 119:259–64. doi: 10.1309/GRH4JWX6J9J7QQTA 12579997

[39] Chamberlain BK McClain CM Gonzalez RS Coffin CM Cates JMM. Alveolar soft part sarcoma and granular cell tumor: an immunohistochemical comparison study. Hum Pathol. (2014) 45:1039–44. doi: 10.1016/j.humpath.2013.12.021 24746209

[40] Ghannam SM Carter GJ Villatoro TM Berg WA. Granular Cell Tumor of the Breast: Radiologic–Pathologic Correlation. J Breast Imaging. (2021) 3:473–81. doi: 10.1093/jbi/wbab041 38424797

[41] Liu K Madden JF Olatidoye BA Dodd LG. Features of Benign Granular Cell Tumor on Fine Needle Aspiration. Acta Cytol. (2011) 43:552–7. doi: 10.1159/000331146 10432874

[42] Stemm M Suster D Wakely PE Jr Suster S. Typical and Atypical Granular Cell Tumors of Soft Tissue: A Clinicopathologic Study of 50 Patients. Am J Clin Pathol. (2017) 148:161–6. doi: 10.1093/ajcp/aqx058 28898987

[43] Gayen T Das A Shome K Bandyopadhyay D Das D Saha A. Granular Cell Tumor: An Uncommon Benign Neoplasm. Indian J Dermatol. (2015) 60:322. doi: 10.4103/0019-5154.156453 26120181 PMC4458968

[44] Kanat O Ozguc H Yalcinkaya U Cubukcu E. A case of granular cell tumor with an interesting clinical course. Indian J Dermatol Venereol Leprol. (2012) 78:193. doi: 10.4103/0378-6323.93643 22421656

[45] Fanburg-Smith JC Meis-Kindblom JM Fante R Kindblom LG. Malignant granular cell tumor of soft tissue: diagnostic criteria and clinicopathologic correlation. Am J Surg Pathol. (1998) 22:779–94. doi: 10.1097/00000478-199807000-00001 9669341

[46] Nelson BL. Benign Neoplasms of the Oral Cavity. In: Head and Neck Pathology [Internet]. Elsevier (2019). Available online at: https://linkinghub.elsevier.com/retrieve/pii/B978032347916500008X [Accessed 2025 May 15]. p. 197-218.e2

[47] Tiwary AK Mishra DK. Granular cell tumor: An uncommon tumor of Schwann cell origin. Indian J Dermatopathol Diagn Dermatol. (2016) 3:26. doi: 10.4103/2349-6029.184008

